# Misinterpretations of Significance Testing Results Near the P-Value Threshold in the Urologic Literature

**DOI:** 10.7759/cureus.41556

**Published:** 2023-07-08

**Authors:** Pranay R Manda, Manish Kuchakulla, Gabrielle Hochu, Pranav Mudiam, Arjun Watane, Ali Syed, Armin Ghomeshi, Ranjith Ramasamy

**Affiliations:** 1 Urology, Emory University School of Medicine, Atlanta, USA; 2 Urology, Weill Cornell Medical Center, New York, USA; 3 Urology, The University of Tennessee Health Science Center, Memphis, USA; 4 Data Science, University of California Berkeley, Berkeley, USA; 5 Opthalmology, Yale School of Medicine, New Haven, USA; 6 Opthalmology, Case Western Reserve University School of Medicine, Cleveland, USA; 7 Psychiatry, Florida International University, Herbert Wertheim College of Medicine, Miami, USA; 8 Urology, University of Miami, Miami, USA

**Keywords:** p-value, data, urology, statistical errors, statistics

## Abstract

Background

The outcome of a statistical test is to accept or reject a null hypothesis. Reporting a metric as “trending toward significance” is a misinterpretation of the p-value. Studies highlighting the prevalence of statistical errors in the urologic literature remain scarce. We evaluated abstracts from 15 urology journals published within the years 2000-2021 and provided a quantitative measure of a common statistical mistake-misconstruing the function of null hypothesis testing by reporting “a trend toward significance.”

Materials and methods

We performed an audit of 15 urology journals, looking at articles published from January 1, 2000, to January 1, 2022. A word recognition function in Microsoft Excel was utilized to identify the use of the word “trend” in the abstracts. Each use of the word “trend” was manually investigated by two authors to determine whether it was improperly used in describing non-statistically significant data as trending toward significance. Statistics and data analysis were performed using Python libraries: pandas, scipy.stats, and seaborn.

Results

This study included 101,134 abstracts from 15 urology journals. Within those abstracts, the word “trend” was used 2,509 times, 572 uses of which were describing non-statistically significant data as trending toward significance. There was a statistically significant difference in the rate of errors between the 15 journals (p < 0.01). The highest rate of improper use of the word “trend” was found in Bladder Cancer with a rate of 1.6% (p < 0.01) of articles. The lowest rate of improper use was found in European Urology, with a rate of 0.3% (p < 0.01). Our analysis found a moderate correlation between the number of articles published and the number of misuses of the word "trend" within each journal and across all journals every year (r = 0.61 and 0.70, respectively).

Conclusion

The overall rate of p-value misinterpretation never exceeded 2% of articles in each journal. There is significance in the difference in misinterpretation rates between the different journals. Authors' utilization of the word “trend” describing non-significant p-values as being near significant should be used with caution.

## Introduction

Statistical errors are common in the medical literature across varied specialties [1-3]. Urology studies are not immune to these errors; a 2005 report found that 71% of sample studies had at least one statistical error [4]. Despite its prevalence, there remains a scarcity of studies highlighting these errors [5-6]. Knowledge of statistical methods is essential for the urologist - both as authors of sound investigations and as readers critically evaluating the literature. In efforts to hold urology accountable for valid reporting, this study is focusing on misunderstanding the *p*-value by using it to report a trend toward significance.

The *p*-value is the probability of finding the observed result when the null hypothesis is true. Study authors establish the probability of committing a type one error or α, and if the *p*-value is less than α, the null hypothesis is rejected. p-values greater than the preset α are reported as nonsignificant.

However, the last two decades have seen a rise in reporting of “almost” significant findings, utilizing expressions such as “trend toward significance” [7]. This practice, while likely unintentional by study authors, is wrong and displays a misunderstanding of the *p*-value. Doing so implies that there is an “almost rejected” null hypothesis category; however, the purpose of applying inferential statistics is to reject or fail to reject the null hypothesis. This practice brings subjectivity and biased reporting to what ought to be objective scientific reporting.

Our objective is to provide a quantitative measure of the aforementioned error in urologic literature. To do this, we examined the frequency of the use of the word "trend" to describe almost significant data in abstracts of urologic literature from 2000 to 2021. While other specialties have estimated the frequency of this error in their body of research, to our knowledge, this is the first study in the urology literature [8-10].

This article was previously presented as a poster at: https://www.auajournals.org/doi/10.1097/JU.0000000000003329.

## Materials and methods

We performed an audit of 15 urology journals, looking at articles published from January 1, 2000, to January 1, 2022. For journals that started publishing after the start of our collection period, all articles from conception to present were included in the analysis. Using PubMed, a total of 101,134 eligible abstracts were extracted. A word recognition software was utilized to identify the use of the word “trend” in the abstracts. Additionally, we searched for "almost significant," "near significant," and "near significance." However, these terms did not add to our abstract pool. Each of the 2,509 uses of the word "trend" was manually scrutinized by two authors and was evaluated whether it was improperly used in describing non-statistically significant data as trending toward significance. What we qualified as improper use of the phrases was describing data as trending toward significance in scenarios where the p-value is listed as greater than 0.05; when a given confidence interval included the value “1.0” (i.e. CI=0.65-1.08); and when the authors directly mention that the data did not reach statistical significance. Statistics and data analysis were performed using Python libraries: pandas, scipy.stats, and seaborn. Three correlation coefficients were calculated: the number of articles published by each journal each year vs. the number of errors in each journal each year; the number of articles in all journals each year vs. the number of errors in all journals each year; and the proportion of errors in all journals each year vs. the number of articles in all journals each year. An H-test was conducted to evaluate the significance of variance between the error statistics across journal populations.

## Results

**Figure 1:**
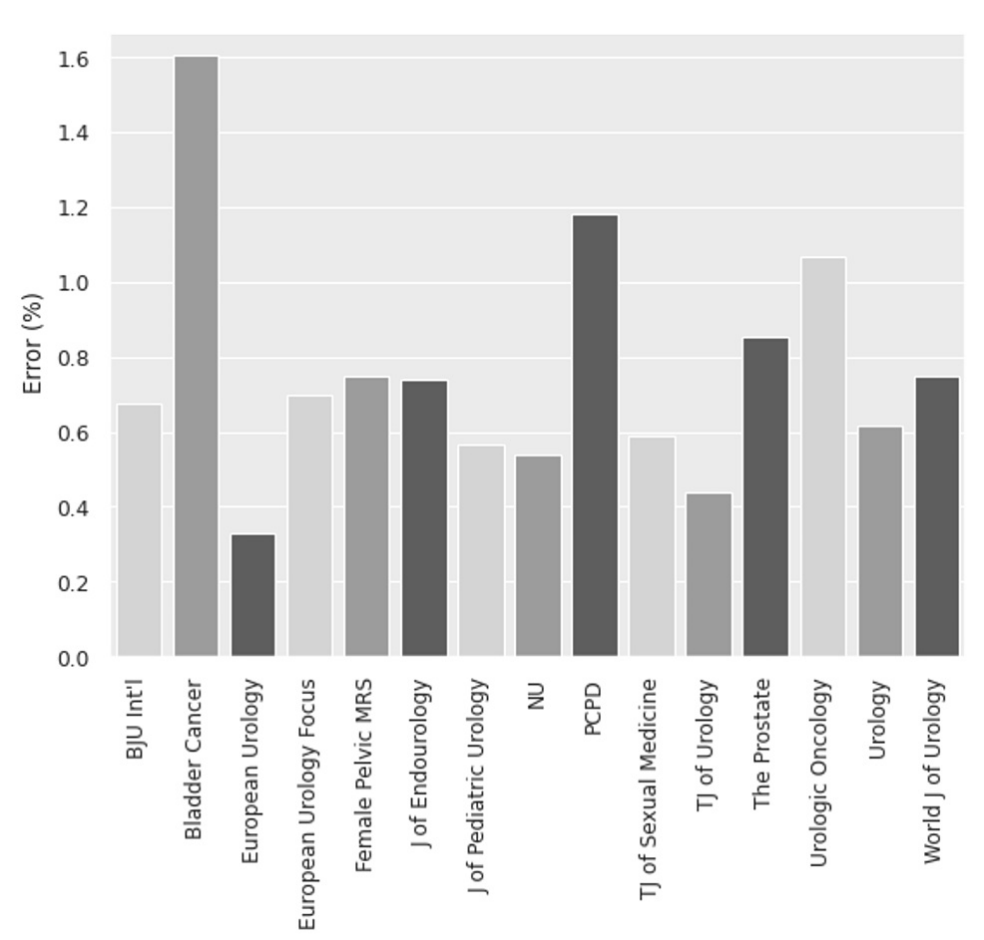
Percentage of articles within years 2000-2021 that used the word “trend” to describe data as near significant in journals

**Figure 2:**
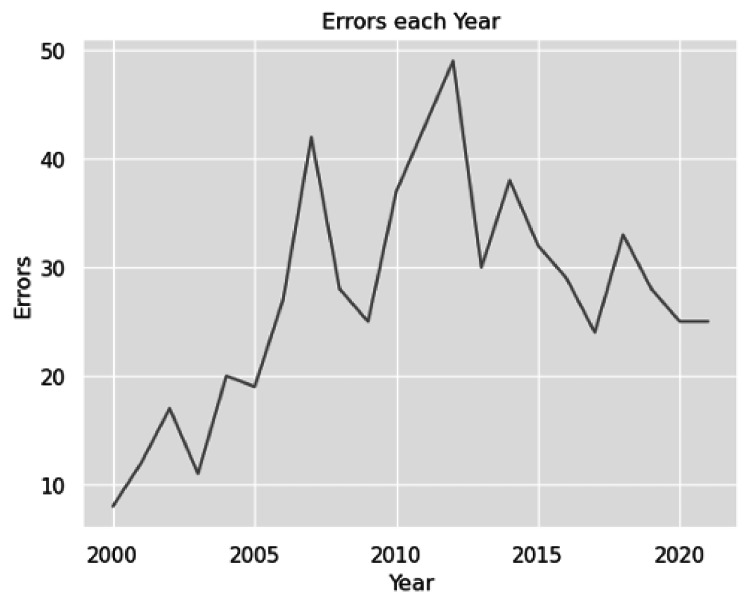
Number of errors using the word “trend” to describe data as near significant each year

**Figure 3:**
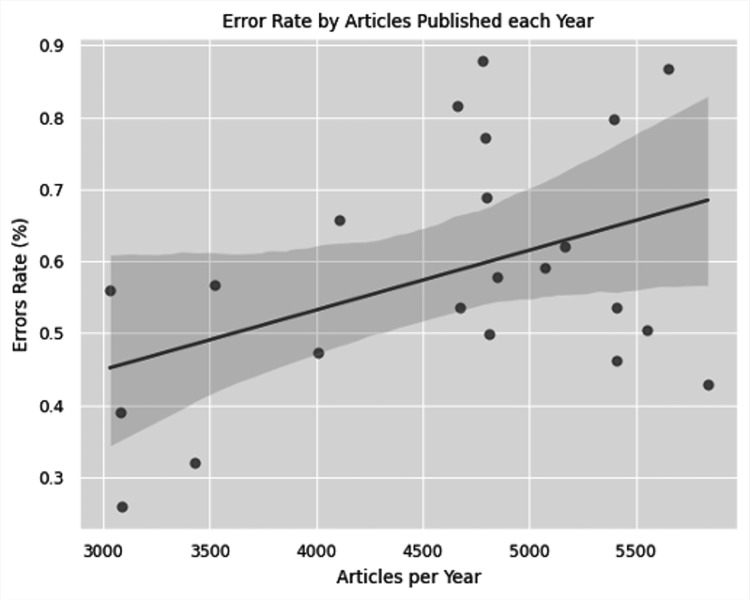
Linear regression plot looking at the proportion of errors and number of yearly articles published (r = 0.43)

This study included 101,134 abstracts from 15 urology journals. Within those abstracts, the word “trend” was used 2,509 times. Of the uses of the word “trend,” 572 uses were describing non-statistically significant data as trending toward significance. There was a statistically significant difference in the rate of errors between the 15 journals (p < 0.01). The highest rate of improper use of the word “trend” was found in Bladder Cancer with a rate of 1.6% (p < 0.01) of articles. The lowest rate of improper use was found in European Urology with a rate of 0.3% (p < 0.01) (Table 1 and Figure 1). Over the years 2000-2021, there is an overall increasing number of errors in published abstracts (Figure 2). Our analysis found a moderate correlation between the number of articles published every year and the number of improper uses of the word trend in each individual journal and across all journals (r = 0.61 and 0.70, respectively). Finally, we found a weak-moderate correlation in the proportion of errors with increasing publications with an r-value of 0.43 (Figure 3).

**Table 1:** Error rates in urology journals

Journal	Papers Published	Papers with Errors	Error Rate
BJU International	11,516	78	0.68%
Bladder Cancer	187	3	1.60%
European Urology	10,296	34	0.33%
European Urology Focus	1,428	10	0.70%
Female Pelvic MRS	1,336	10	0.75%
J of Endourology	5,681	42	0.74%
J of Pediatric Urology	3,876	22	0.57%
Neurourology and Urodynamics	3,910	21	0.54%
Prostate Cancer and Prostatic Diseases	1,607	19	1.18%
TJ of Sexual Medicine	5,097	30	0.59%
TJ of Urology	26,817	118	0.44%
The Prostate	3,747	32	0.85%
Urologic Oncology	4,116	44	1.07%
Urology	16,696	103	0.62%
World J of Urology	4,823	36	0.75%

## Discussion

The outcome of a statistical test is to accept or reject a null hypothesis. Reporting a metric as “trending toward significance” is a misinterpretation of the p-value. To provide a quantitative measure of this error in the urologic literature, we examined the frequency of this statistical error from 2000 to 2020 in 15 urology journals. Our study found a statistically significant difference in the rate of errors among the journals included. Bladder Cancer was found to have the highest rate of p-value misinterpretation with a rate of 1.6% over the lifetime of the journal and European Urology with the lowest rate of error at 0.3% (Table 1). We found a moderate linear correlation between the number of articles each year and the number of errors among all journals and within each individual journal. Additionally, we identified a weak-moderate correlation in the proportion of errors with increasing publications with an r-value of 0.43 (Figure 3). These findings are important to highlight because they suggest that some urologic literature gives meaning to statistical analysis when none is present.

There is a paucity of literature looking into statistical errors in academic medical journals, especially in the field of urology. To the best of our knowledge, no prior studies have evaluated the rate of statistical errors within the urologic literature. An error rate of 1.02% within six major neurosurgery journals was found, similar to the rate in our study [8]. Some authors suggest that the p-value inappropriately dichotomizes data as significant or insignificant, creating a false sense of confidence in data that reach the p-value threshold. These authors suggest adopting the use of an alternative “surprise” or “s-value” that converts a p-value into the number of successive identical results of flips of a fair coin [11]. However, no clear alternative has been adopted as a standard of practice at this time.

It is difficult to postulate a specific reason for the discrepancy in errors between the journals. An important factor to highlight within the urologic literature specifically is that, in 2018, four journals, namely, European Urology, BJU International, Urology, and the Journal of Urology, all published guidelines regarding the reporting of statistics to improve the quality of the data reported [12]. These guidelines specifically advise against reporting p-values >0.05 as “trending” toward significance. It would be reasonable to expect an improvement in the rate of misinterpretation following this change in the coming years. All aspects considered, it is likely that most of these statistical errors are unintentional and a result of insufficiency in statistical method education. For future guidance, authors should utilize caution when using the word “trend” and avoid misinterpreting the p-value. Journals should consider following the steps BJUI, European Urology, Urology, and Journal of Urology have taken to minimize these errors by publishing guidelines on the reporting of statistics.

As with most studies, there are limitations to our analysis. This analysis likely underestimated the number of p-value misinterpretations for two reasons. The first reason is that only abstracts were analyzed as opposed to full-body texts. It is likely errors would have been discovered within the body of several more papers. Additionally, our main search criteria in searching for errors were reliant on the use of the word “trend.” While this likely covers many misinterpretations of p-values, it cannot be proven that similar errors were not made without the use of the word trend. Additionally, we attempted to elicit a broad range of urologic literature by including 15 journals in our analysis; however, this does not cover all the urologic literature that exists. 

## Conclusions

There is an overall increasing number of errors in describing data with p-values near the significance threshold as “trending toward significance.” The highest proportion of this error was found in Bladder Cancer, and the lowest proportion was found in European Urology. The reason these errors are made is likely multi-factorial and unintentional. Recommendations for cutting down such errors would include stricter guidelines for the reporting of statistics and heavier scrutiny by reviewers regarding how data are presented. Publishing guidelines for statistics can help educate authors regarding the proper reporting of statistics and help set expectations. This paper aims to bring light to these errors and take a step forward in preventing future misinterpretations of the p-value in the urologic literature. Both reviewers and authors should be wary about the use of the word trend and ensure that it is used properly when describing data.
